# Supplementary material for: Layer-dependent exciton polarizability and the brightening of dark excitons in few-layer black phosphorus
Source: Nat Commun. 2023 Sep 1;14:5314. doi: 10.1038/s41467-023-41126-8 (PMC10474117; doi:10.1038/s41467-023-41126-8)
Supplement: Supplementary file 1 — Supplementary Information [file 41467_2023_41126_MOESM1_ESM.pdf]

Supplementary Information for
Layer-dependent exciton polarizability and the brightening of dark excitons in few-layer black phosphorus

Yuchen Lei[1], Junwei Ma[1], Jiaming Luo[1], Shenyang Huang[1], Boyang Yu[1], Chaoyu Song[1], Qiaoxia Xing[1], Fanjie Wang[1], Yuangang Xie[1], Jiasheng Zhang[1], Lei Mu[1], Yixuan Ma[1], Chong Wang[2,3] & Hugen Yan*[1]

[1] State Key Laboratory of Surface Physics, Key Laboratory of Micro and Nano-Photonic Structures (Ministry of Education), and Department of Physics, Fudan University, Shanghai 200433, China.

[2]Centre for Quantum Physics, Key Laboratory of Advanced Optoelectronic Quantum Architecture and Measurement (MOE), School of Physics, Beijing Institute of Technology, Beijing, 100081, China

[3]Beijing Key Lab of Nanophotonics & Ultrafine Optoelectronic Systems, School of Physics, Beijing Institute of Technology, Beijing, 100081, China

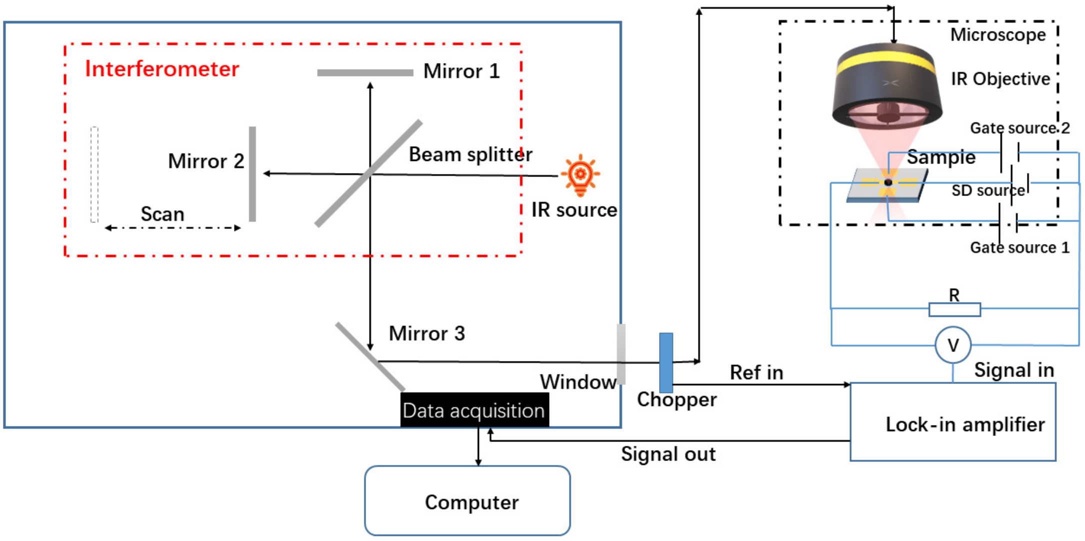

Supplementary Figure 1 | The setup of the spectrum-resolved photocurrent measurement. The left part in the blue box represents the hardware inside FTIR main chamber. The right part is the experiment configuration associated with the microscope attached.

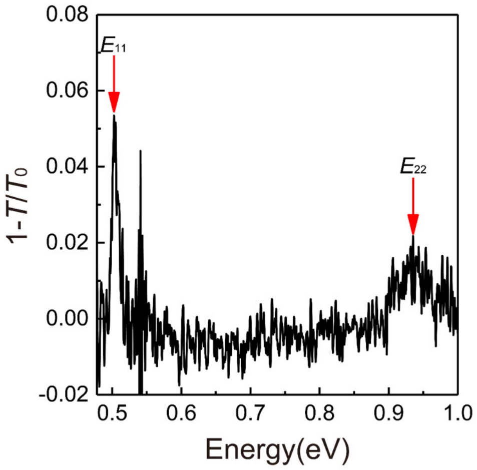

Supplementary Figure 2 | The extinction spectrum of the sample mentioned in Fig.

1c with light polarization along the armchair direction of BP. The extracted peak positions are 0.502 eV and 0.935 eV respectively, in good agreement with the normalized photocurrent spectrum in which peak positions are 0.512 eV and 0.931 eV respectively.

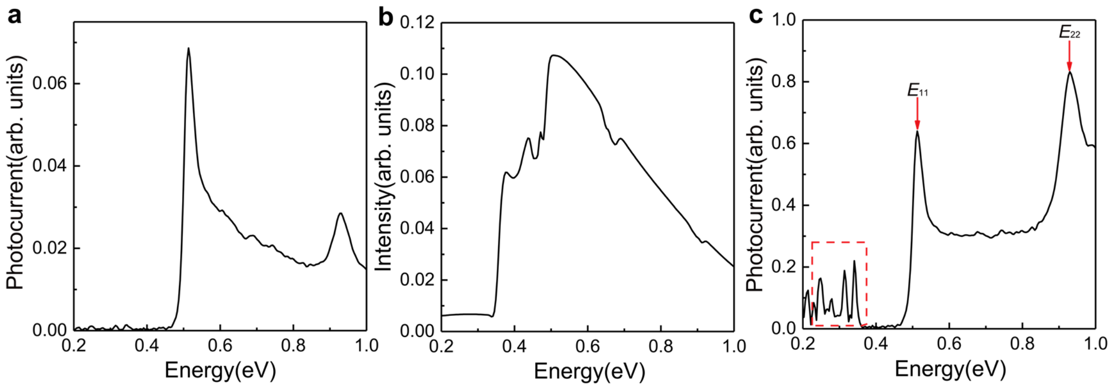

Supplementary Figure 3 | The normalization process of the photocurrent spectrum for an ungated 7-layer sample. a) The original photocurrent spectrum. b) The reflection spectrum of illumination system measured on a gold mirror. c) The normalized photocurrent spectrum of Fig. 2a, obtained through the spectrum in panel a divided by the spectrum in panel b. The spikes in the red dashed rectangle area are caused by the diminishing light intensity at this energy region, as shown in panel b.

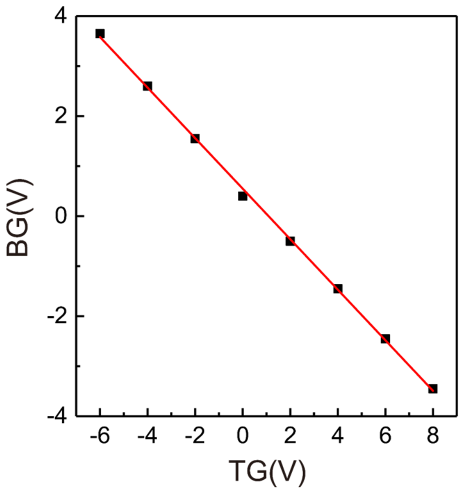

Supplementary Figure 4 | The back-gate voltage for the charge neutral point as a function of the top-gate for the 6-layer sample shown in Fig. 2.

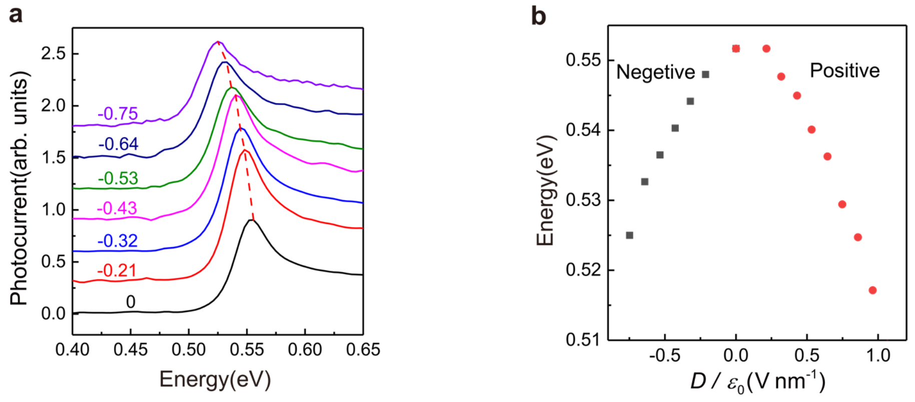

Supplementary Figure 5 | The normalized photocurrent spectrum under opposite vertical electric fields. a) The normalized photocurrent spectrum of the 6-layer BP device under a series of electrical fields from $0\ \mathrm{V\,nm^{-1}}$ to $-0.75\ \mathrm{V\,nm^{-1}}$. Spectra are shifted vertically for clarity. b) The E_{11} positions extracted from Fig. 2c and Supplementary Fig. 5a as a function of the external field.

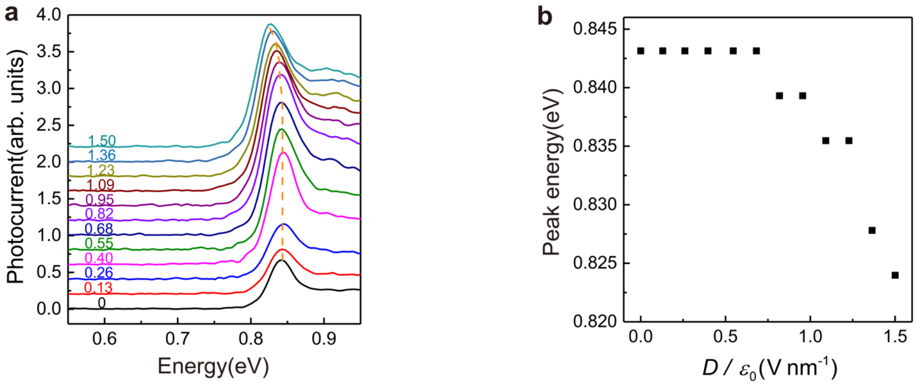

Supplementary Figure 6 | The QCSE of a 3-layer BP. a) The normalized photocurrent spectra of a 3-layer BP FET under a series of electrical fields from $0\ \mathrm{V\,nm^{-1}}$ to $1.89\ \mathrm{V\,nm^{-1}}$. The orange dashed line indicates the E_{11} peak evolution. Spectra are vertically shifted for clarity. **b)** The peak positions of E_{11} extracted from Supplementary Fig. 6a.

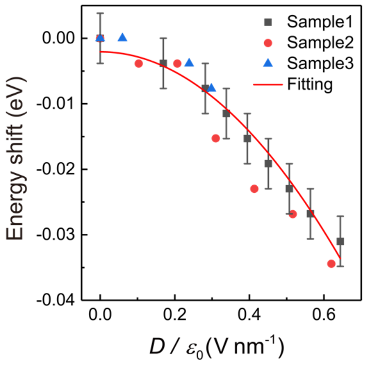

Supplementary Figure 7 | The E_{11} energy shifts extracted from three different 9-layer BP devices. The red curve represents the quadratic fitting result of all the points. We added error bars for sample1 data points. The figure shows that QCSE is

highly reproducible (within the experimental uncertainty) for the same thickness samples.

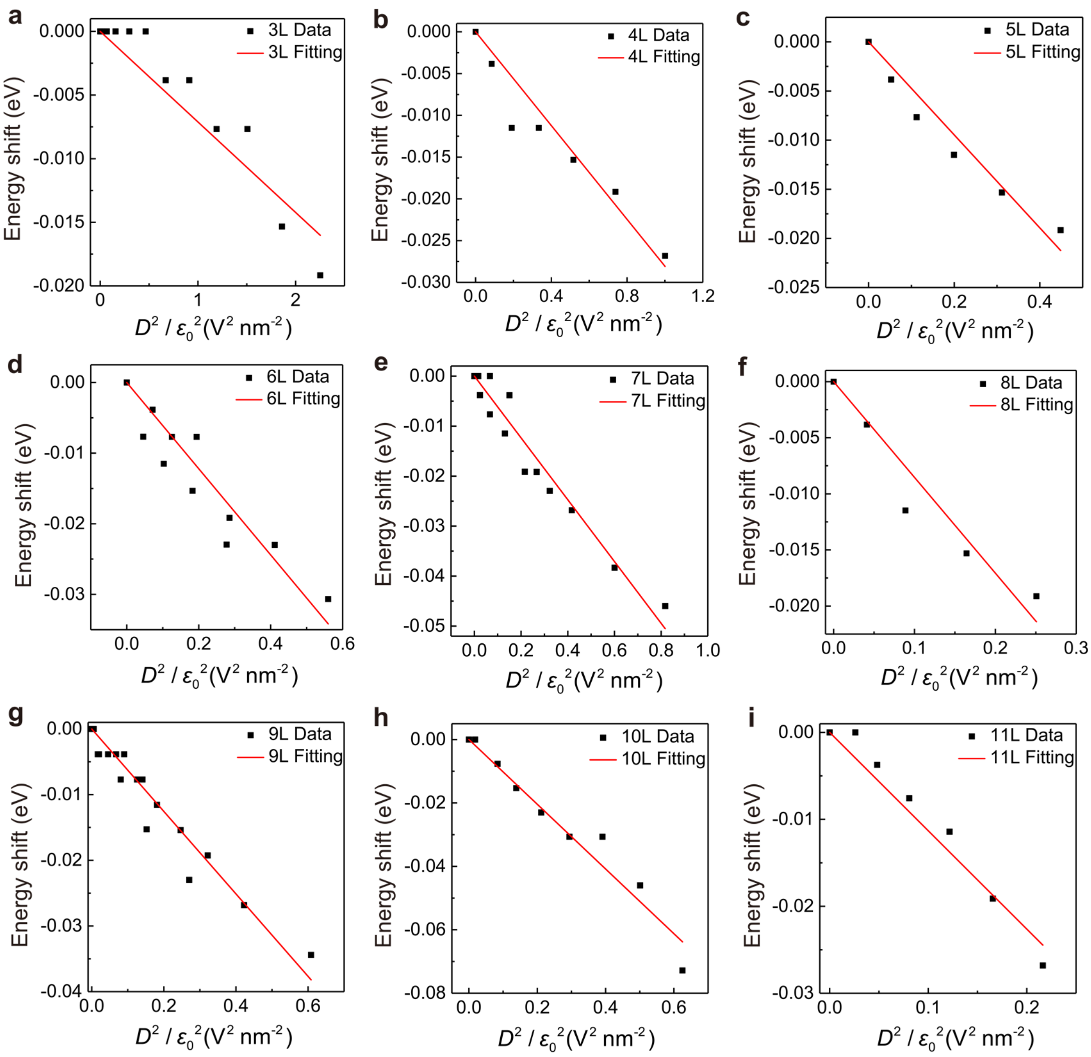

Supplementary Figure 8 | The extraction of the polarizabilities (α). **a-i)** The fitting results of 3- to 11-layer BP devices. The polarizability α and the quality factor R^2 are shown in the table below.

Layer number	Polarizability α (eV nm^2 V^{-2})	R^2
3	0.0071±0.0006	0.935
4	0.0285±0.0020	0.969
5	0.0417±0.0039	0.966
6	0.0480±0.0041	0.889
7	0.0618±0.0028	0.974
8	0.0853±0.0075	0.970
9	0.0918±0.0021	0.989
10	0.1021±0.0050	0.980
11	0.1212±0.0065	0.980

Supplementary Table 1 | The fitting result of Supplementary Fig. 8

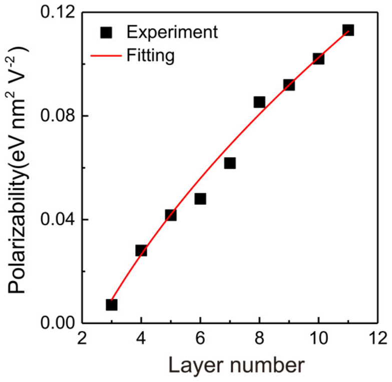

Supplementary Figure 9 | A tentative fitting of the layer-dependence of the experimental polarizabilities (α). We fitted them against layer number L with a phenomenological polynomial: $\alpha = A + B \cdot L^C$ and got $C = 0.502$.

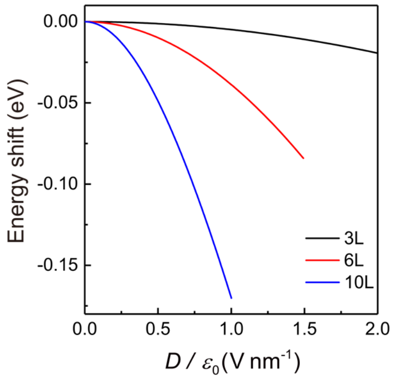

Supplementary Figure 10 | The QW model calculations of the E_{11} shift under external field in three BP samples with different thickness. The method of calculation is detailed in Supplementary Note 2. It is clear that the shift of E_{11} increases with the thickness.

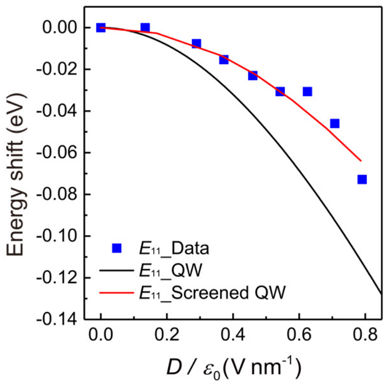

Supplementary Figure 11 | The comparison between QW calculation, screened QW calculation and experimental data of the E_{11} shift in a 10-layer BP FET.

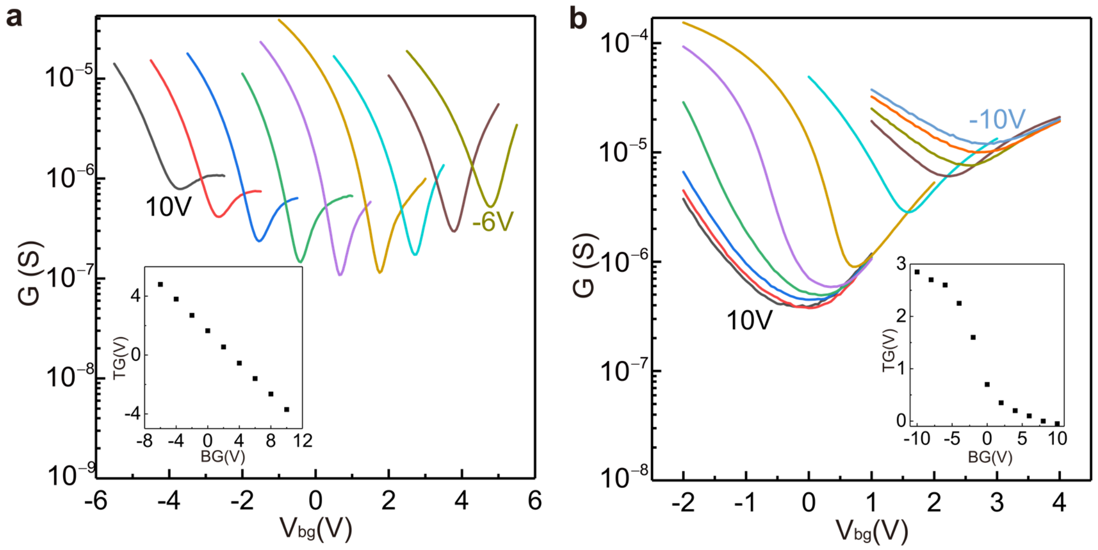

Supplementary Figure 12 | Comparison of the transport measurement of BP devices with two thicknesses. **a)** The transfer curves of a 13-layer BP device. The top gate is tuned discretely from -6.0 V to 10 V while the bottom gate is swept continuously. The inset shows the extracted positions of the charge neutral point. The shift is linear.

b) The transfer curves of a 20-layer BP device. The inset shows the extracted charge neutral points. It is apparent that the shift is non-linear, indicating the presence of free carrier screening[1].

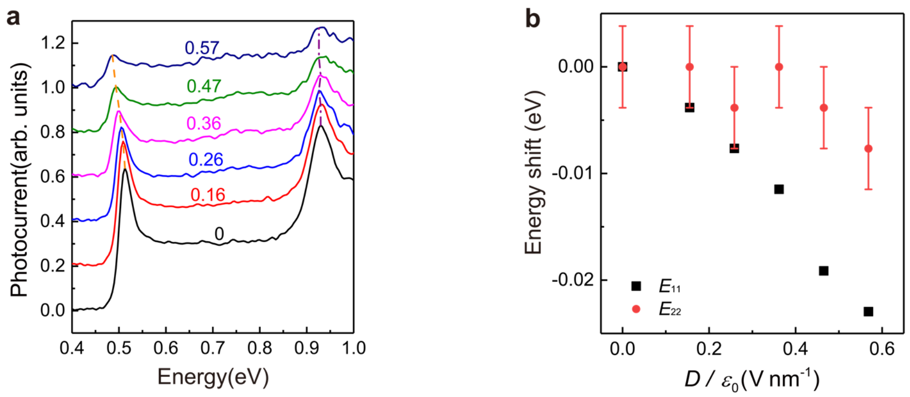

Supplementary Figure 13 | The photocurrent spectra of a 7-layer BP under increasing vertical electric field at room temperature. **a)** The photocurrent spectra under increasing field. The unit of the labels in the figure is V nm^{-1}.The orange dashed line and the violet dot dashed line represent the evolution of E_{11} position and E_{22} position respectively. Spectra are vertically shifted for clarity. **b)** The peak shifts of E_{11} and E_{22} extracted from Supplementary Fig. 13a. The error bars of the E_{22} data points are included, which originate from the measurements of the spectrum.

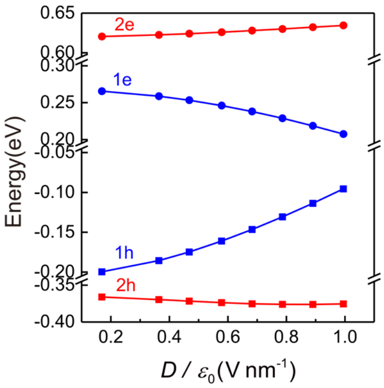

Supplementary Figure 14 | The index-dependent screened QW calculation for a 10-layer BP sample. The absolute values of the band energies are imprecise because the electric potential energy is not considered, but the shift trends are accurate.

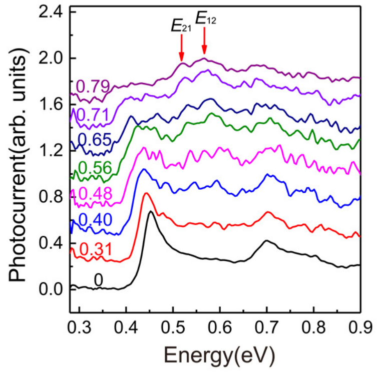

Supplementary Figure 15 | The photocurrent spectra at room temperature for the 10-layer BP studied in Fig. 4. The unit of the labels in the figure is $\mathrm{V\,nm^{-1}}$. The red arrows point out the newly emerged features representing E_{21} and E_{12} respectively. Spectra are vertically shifted for clarity.

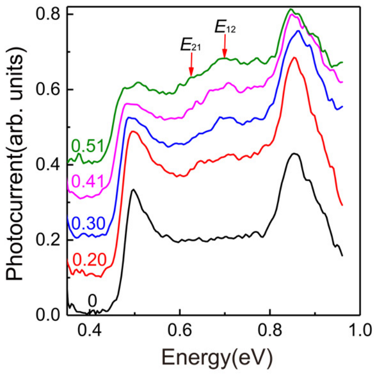

Supplementary Figure 16 | The photocurrent spectra of an 8-layer BP device at room temperature. The unit of the labels in the figure is $\mathrm{V\,nm^{-1}}$. The red arrows point out the newly emerged features which represent the forbidden transitions E_{21} and E_{12} respectively. Spectra are vertically shifted for clarity.

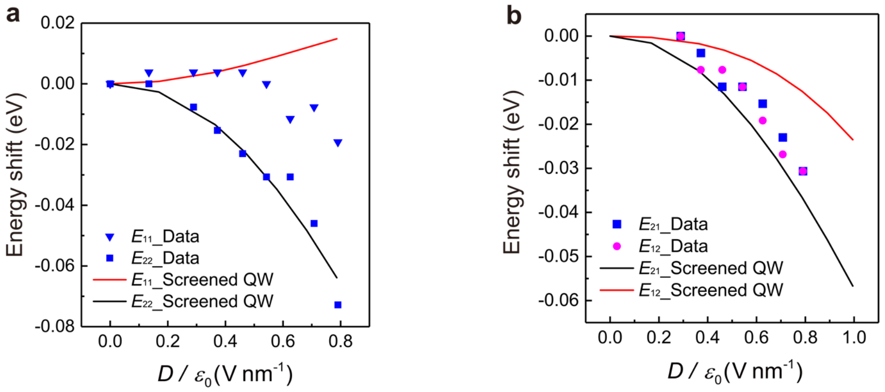

Supplementary Figure 17 | The experimental data and the screened QW calculations of the transitions in the 10-layer BP sample. **a)** Peak energy shifts of the E_{11} and E_{22} transitions. The solid curves represent calculations based on screened QW theory. The blue dots are the experimental data extracted from Fig. 4a. **b)** Peak energy shifts of

the forbidden transitions E_{21} and E_{12}. The solid curves represent calculations based on screened QW theory. The blue squares and violet circles are the experimental data extracted from Fig. 4a.

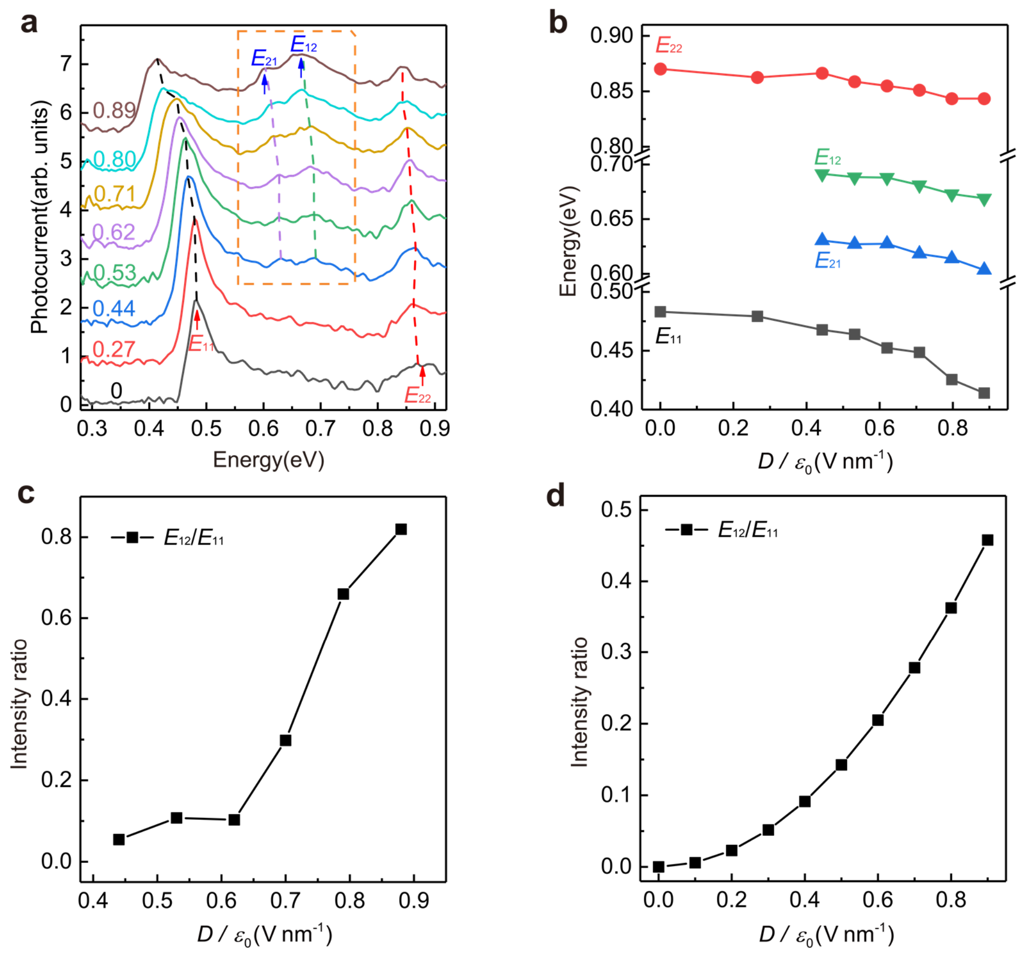

Supplementary Figure 18 | The field-dependent photocurrent spectra of an 8-layer BP. **a)** The normalized photocurrent spectra of an 8-layer BP device measured at 77.5 K under a series of external fields. The unit of the numerical labels for the field in the figure is $\mathrm{V\,nm^{-1}}$. The orange dashed frame highlights the emerging E_{21} and E_{12} transitions, and their positions are guided by dashed lines. The black and red

dashed lines indicate the evolution of the E_{11} peak and the E_{22} peak respectively. Spectra are vertically shifted for clarity. **b)** The peak positions extracted from Supplementary Fig. 18a as a function of external field. **c)** The intensity ratio of E_{12} to E_{11} transitions extracted from Supplementary Fig. 18a as a function of field. The intensities were extracted by Lorentz fitting of the peaks in the spectra. **d)** Numerical calculation of the corresponding intensity ratio.

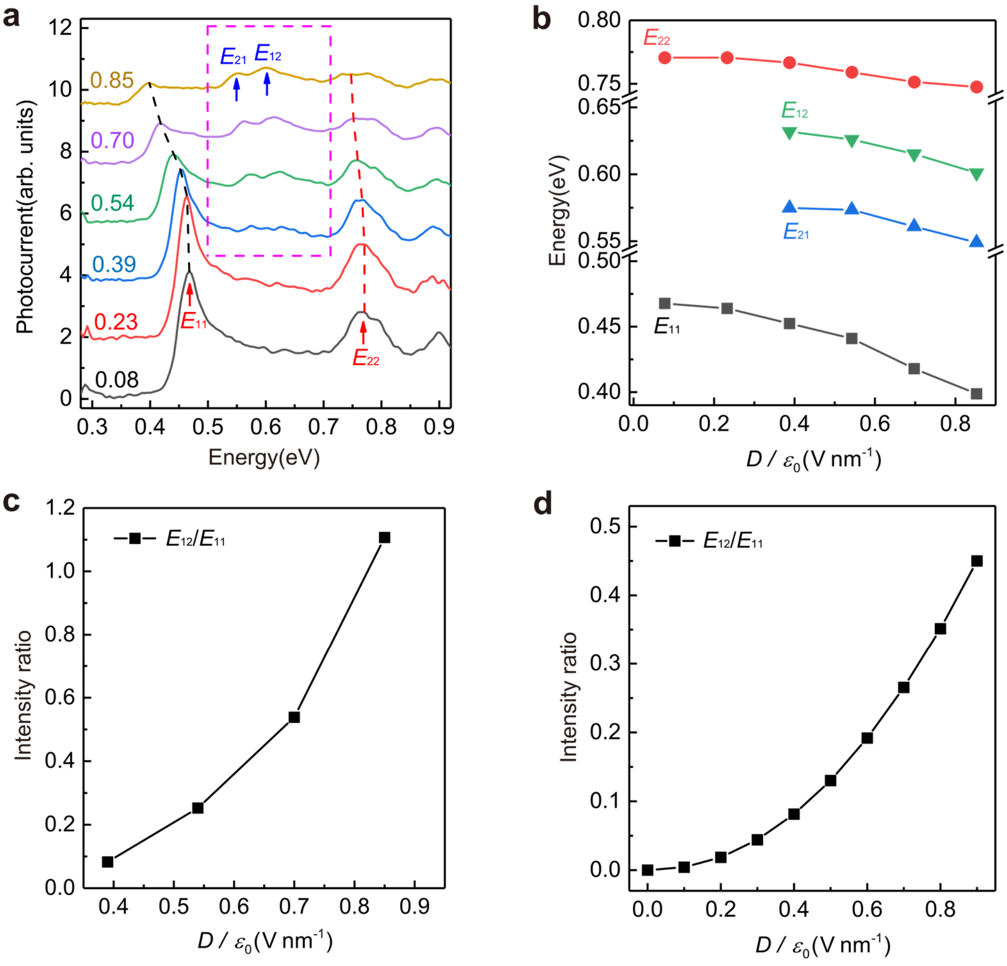

Supplementary Figure 19 | The field-dependent photocurrent spectra of a 9-layer BP.

a) The normalized photocurrent spectra of a 9-layer BP device measured at 77.5 K under a series of external fields. The unit of the numerical labels for the field in the figure is $\mathrm{V\,nm^{-1}}$. The magenta dashed frame highlights the emerging E_{21} and E_{12} transitions. The black and red dashed lines indicate the evolution of the E_{11} peak and the E_{22} peak respectively. Spectra are vertically shifted for clarity. **b)** The peak positions extracted from Supplementary Fig. 19a as a function of external field. **c)** The intensity ratio of E_{12} to E_{11} transitions extracted from Supplementary Fig. 18a as a function of field. The intensities were extracted by Lorentz fitting of the peaks in the spectra. **d)** Numerical calculation of the corresponding intensity ratio.

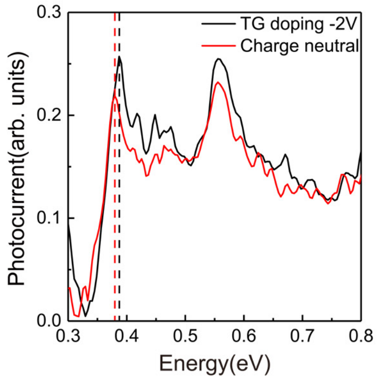

Supplementary Figure 20 | Single top-gate spectra of a 13-layer BP measured at 77.5 K. The blueshift (rather than redshift) of the E_{11} peak indicates the dominance of Burstein Moss Shift (BMS) over QCSE.

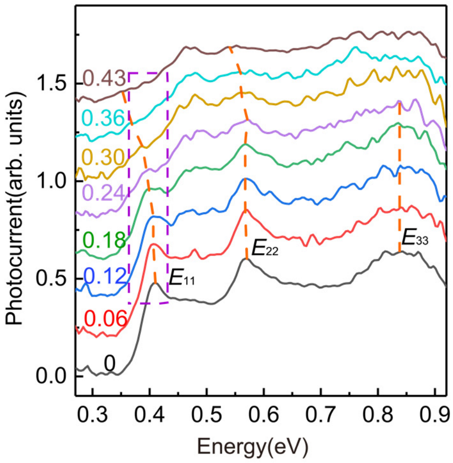

Supplementary Figure 21 | The photocurrent spectrum of a 13-layer BP measured at room temperature. The orange dashed lines outline the positions of E_{11}, E_{22} and E_{33} excitons. The violet dashed frame highlights the rapidly diminishing E_{11} peak.

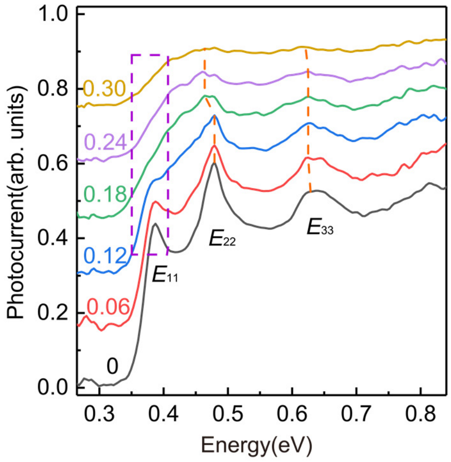

Supplementary Figure 22 | The photocurrent spectrum of an 18-layer BP measured at room temperature. The orange dashed lines outline the positions of E_{22} and E_{33} excitons. The violet dashed frame highlights the rapidly diminishing E_{11} peak.

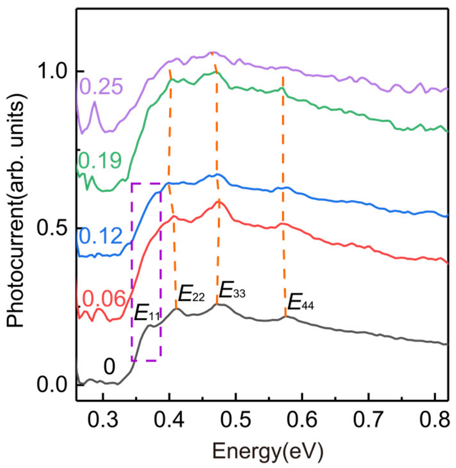

Supplementary Figure 23 | The photocurrent spectrum of a 20-layer BP measured at room temperature. The orange dashed lines outline the positions of E_{22}, E_{33} and E_{44} excitons. The violet dashed frame highlights the rapidly diminishing E_{11} peak.

Supplementary Note 1

The procedure of normalization for the spectra

The raw photocurrent spectrum obtained from the FTIR spectrometer cannot truly represent the absorption, because the light source energy is not uniform across the whole spectrum. For example, in Supplementary Fig. 3a between the two main peaks we can see an obvious decline in the photocurrent with increasing photon energy, which actually originates from the declining intensity of the light source in that spectral range. Hence, we need to normalize the spectrum to eliminate the source impact. Spectrum of the light source itself was obtained through reflection measurement from a gold mirror, as shown in Supplementary Figure 3b. Then, the

normalization was performed through dividing raw photocurrent spectrum (shown in Supplementary Fig. 3a) by light source spectrum (shown in Supplementary Fig. 3b), obtaining the normalized photocurrent spectrum as shown in Supplementary Fig. 3c. The spikes in the red dashed frame are caused by the dramatically enhanced noise due to the lack of light intensity at that energy range.

Supplementary Note 2

A one-dimensional infinite quantum well under an electric field

First we try to approximate the hBN/PB/hBN device as a one-dimensional infinite QW to estimate the band shift, as shown in Supplementary Fig. 24. For the convenience of subsequent calculations, we choose to set the origins of z-axis and of electrostatic potential at the center of the well (thickness of L). In this case, the Hamiltonian of the system is

$$\hat{\mathcal{H}} = -\frac{\hbar^2}{2m_z}\frac{\mathrm{d}^2}{\mathrm{d}z^2} + V_{\mathrm{conf}}(z) - eEz. \quad \text{(S1)}$$

E is the electric field, which is expressed as $E = D/\varepsilon_0\varepsilon_{\mathrm{BP}}$, where $\varepsilon_{\mathrm{BP}}$ is the relative permittivity in the z-direction of BP and depends on the number of layers, as shown in Table. S2[2].

V_{conf} is the well potential with the following expression

$$V_{\mathrm{conf}}(z) = \begin{cases} 0, & -L/2 \le z \le L/2 \\ \infty, & \text{else} \end{cases}, \quad \text{(S2)}$$

m_z is the effective mass in the z-direction, which is $0.128m_0$ for electrons and $0.280m_0$ for holes[3]. Due to the lack of relevant research on the effective mass in the z-direction of few-layer BP, here we use the effective mass of the bulk BP.

Correspondingly, the Schrodinger equation inside the well can be written as:

$$-\frac{\hbar^2}{2m_z}\frac{\mathrm{d}^2}{\mathrm{d}z^2}\Psi(z)-(\mathcal{E}+eEz)\Psi(z)=0 \tag{S3}$$

where $\mathcal{E}$ is the eigenenergy. Then we can further simplify this equation by introducing a dimensionless variable[4,5] :

$$Z=-\left[\frac{2m_z}{(e\hbar E)^2}\right]^{\frac{1}{3}}(\mathcal{E}+eEz)\,. \tag{S4}$$

Therefore, Eq. (S3) can be simplified to

$$\frac{\mathrm{d}^2}{\mathrm{d}Z^2}\widetilde{\Psi}(Z)-Z\widetilde{\Psi}(Z)=0 \tag{S5}$$

with the lth solution consisting of Airy functions $\mathrm{Ai}(Z)$ and $\mathrm{Bi}(Z)$:

$$\widetilde{\Psi}_l(Z)=C_{1l}\mathrm{Ai}(Z)+C_{2l}\mathrm{Bi}(Z) \tag{S6}$$

where C_{1l} and C_{2l} are the coefficients to be determined. With the use of the boundary conditions:

$$\widetilde{\Psi}_l\left(Z|_{z=\pm L/2}\right)=0 \tag{S7}$$

we can get the equation that eventually needs to be solved numerically as:

$$\mathrm{Ai}\left(Z|_{z=+L/2}\right)\mathrm{Bi}\left(Z|_{z=-L/2}\right)-\mathrm{Ai}\left(Z|_{z=-L/2}\right)\mathrm{Bi}\left(Z|_{z=+L/2}\right)=0\,, \tag{S8}$$

and its solution will give the shifts of energy levels with the electric field. We calculated the shifts of v_1 and c_1 bands and eventually E_{11} transitions of 3- to 8-layer BP versus the field with this model. The results for 3-layer and 6-layer samples are shown in Fig. 3a. Combining the eigenenergy with (S4), (S6), (S7), the wave functions corresponding to different eigenenergies can be obtained as well.

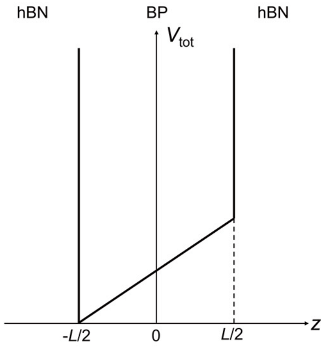

Supplementary Figure 24 | Schematic diagram of the QW model. The vertical axis represents the potential energy of electrons. The black thick solid line in the figure represents the one-dimensional infinite QW in an electric field.

Layer number	Relative permittivity
3	3.5
4	4.1
5	4.5
6	4.9
7	5.2
8	5.5
9	5.7

10	5.9
11	6.1

Supplementary Table 2 | Relative permittivity in the z-direction of BP with different thickness

Supplementary Note 3

The physical mechanism of the index dependent QCSE.

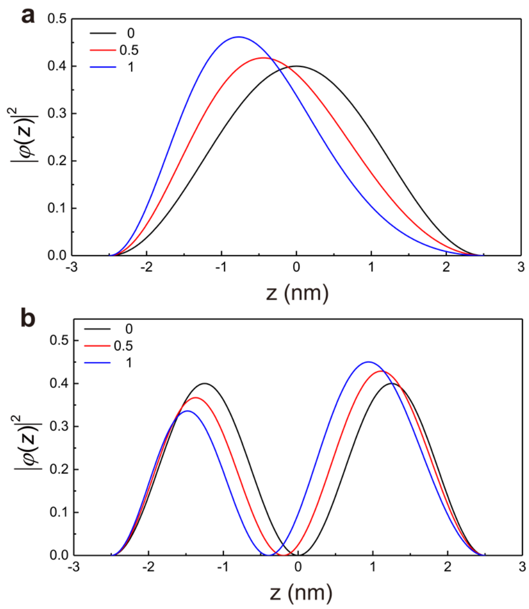

Supplementary Figure 25 | The calculated wave functions of electrons under moderate electric fields. The unit of the numerical labels for the field in the figure is V nm^{-1}. a) The calculated wave functions of $l = 1$ electrons. b) The calculated wave functions of $l = 2$ electrons.

Supplementary Figure 25 displays the calculated wave functions of electrons using the method mentioned in Supplementary Note 2. Under the applied field, the closer the wave function of electrons is to the boundary of the QW on the lower electric potential side, the lower the energy, while the opposite is true for holes. We can see that under electric fields the wave function of the lowest $l = 1$ state has a larger amplitude in the negative region of z compared with the ungated ones, inferring the reduction of energy. On the other hand, although for $l = 2$ states the wave function moves to the negative z direction as a whole, there is still a relatively large amplitude in the $z > 0$ region. This even yields a positive energy shift for $l = 2$ states[5], which could drag the shift pace down when the field is moderate. As a result, the E_{22} transition shifts more slowly than the E_{11} transition in the same sample.

Supplementary Note 4

Screening effect based on the Thomas-Fermi model and the band structure

Here we focus on using a nonlinear Thomas-Fermi (TF) model including interlayer hopping to evaluate the screening properties of multilayer BP films. For Thomas-Fermi model in a more general case, one can refer to Ref [6-10]. It should also be pointed out that in subsequent calculations we choose to set the origin of z-axis at the bottom surface of the BP film, which is different from the previous one.

We assumed that two parallel plates with net surface-charge density $-en_0$ and en_0 respectively attached to either side of the BP film generate the applied electric field. Both free electrons and holes that play the role of screening in the BP film mainly come from thermal excitation, as shown in Supplementary Fig. 26. Due to the presence of the applied field, the thermally excited carrier density in the layer at different positions is different, but at the same time the overall film remains electrically neutral according to our conditions chosen from the transfer curve measurements. For a BP film of thickness L, the electron areal number density is $-n_1(z)$ and the hole areal number density is $n_2(z)$ in the BP layer at a distance of z from the positively charged plate. In this case, the grand potential per unit area for this system in the continuum limit is

$$\Omega = \int_0^L \frac{\mathrm{d}z}{d}\{E_\mathrm{k} + E_\mathrm{V1} + E_\mathrm{V2} - \mu[-n_1(z) + n_2(z)]\} \quad \text{(S9)}$$

where d is the interlayer spacing, μ is the chemical potential.

$$E_\mathrm{k} = -A_1 n_1^{\frac{5}{3}}(z) + A_2 n_2^{\frac{5}{3}}(z) \quad \text{(S10)}$$

is the kinetic energy per unit area of electrons and holes of each BP layer, while we have defined $A_1 = (3/10)(6\pi^2\hbar^3/g_s d\sqrt{m_{ex}m_{ey}m_{ez}})^{2/3}$ and $A_2 = (3/10)(6\pi^2\hbar^3/g_s d\sqrt{m_{hx}m_{hy}m_{hz}})^{2/3}$.

$$E_\mathrm{V1} = -\frac{e^2}{2\varepsilon_0\varepsilon_\mathrm{BP}}\{zn_0[n_1(z) + n_2(z)] + (L - z)(-n_0)[n_1(z) + n_2(z)]\} \quad \text{(S11)}$$

is the Coulomb potential per unit area between BP layers and plates, with ε_BP as the dielectric constant of BP film in z-direction. For different thicknesses, it has different

values, as shown in Table. S2[2].

$$E_{\mathrm{V2}} = -\frac{e^2}{2\varepsilon_0\varepsilon_{\mathrm{BP}}}\int_0^L \frac{\mathrm{d}z'}{d}|z-z'|\left[n_1(z)n_2(z') + \frac{1}{2}n_1(z)n_1(z') + \frac{1}{2}n_2(z)n_2(z')\right] \quad (\mathrm{S12})$$

is the Coulomb potential per unit area between BP layers. At the same time, electric neutrality makes $n_1(z)$ and $n_2(z)$ satisfy

$$\int_0^L \frac{\mathrm{d}z}{d}[n_1(z) + n_2(z)] = 0\,. \quad (\mathrm{S13})$$

To minimize Ω, we need to solve the corresponding Euler-Lagrange equations

$$\begin{cases} -f_1(z) + \dfrac{3\mu}{5A_1} + \alpha_1 L n_0 - 2\alpha_1 z n_0 - \alpha_1 \displaystyle\int_0^L \frac{\mathrm{d}z'}{d}|z-z'|\left[-f_1^{\frac{3}{2}}(z') + f_2^{\frac{3}{2}}(z')\right] = 0 \\ f_2(z) - \dfrac{3\mu}{5A_2} + \alpha_2 L n_0 - 2\alpha_2 z n_0 - \alpha_2 \displaystyle\int_0^L \frac{\mathrm{d}z'}{d}|z-z'|\left[-f_1^{\frac{3}{2}}(z') + f_2^{\frac{3}{2}}(z')\right] = 0 \end{cases} \quad (\mathrm{S14})$$

where

$$\begin{cases} f_1(z) = n_1^{2/3}(z) > 0, \text{ with } n_1(z) = -f_1^{3/2}(z) \\ f_2(z) = n_2^{2/3}(z) > 0, \text{ with } n_2(z) = f_2^{3/2}(z) \end{cases} \quad (\mathrm{S15})$$

and we have defined $\alpha_1 = 3e^2/10A_1\epsilon_0\varepsilon_{\mathrm{BP}}$ and $\alpha_2 = 3e^2/10A_2\epsilon_0\varepsilon_{\mathrm{BP}}$. Taking the first derivative of Eqs. (S14) with respect to z gives

$$\begin{cases} -\dfrac{\mathrm{d}f_1(z)}{\mathrm{d}z} - 2\alpha_1 n_0 - \alpha_1 \displaystyle\int_0^L \frac{\mathrm{d}z'}{d}\mathrm{sgn}(z-z')\left[-f_1^{\frac{3}{2}}(z') + f_2^{\frac{3}{2}}(z')\right] = 0 \\ \dfrac{\mathrm{d}f_2(z)}{\mathrm{d}z} - 2\alpha_2 n_0 - \alpha_2 \displaystyle\int_0^L \frac{\mathrm{d}z'}{d}\mathrm{sgn}(z-z')\left[-f_1^{\frac{3}{2}}(z') + f_2^{\frac{3}{2}}(z')\right] = 0 \end{cases} \quad (\mathrm{S16})$$

By combining Eq. (S13), Eqs. (S15) and Eqs. (S16), the boundary conditions of the differential equation system can be obtained

$$\begin{cases} \left.\dfrac{\mathrm{d}f_1(z)}{\mathrm{d}z}\right|_{z=0} = \left.\dfrac{\mathrm{d}f_1(z)}{\mathrm{d}z}\right|_{z=L} = -2\alpha_1 n_0 \\ \left.\dfrac{\mathrm{d}f_2(z)}{\mathrm{d}z}\right|_{z=0} = \left.\dfrac{\mathrm{d}f_2(z)}{\mathrm{d}z}\right|_{z=L} = 2\alpha_2 n_0 \end{cases} \quad (\mathrm{S17})$$

Taking the second derivative of Eqs. (S14) with respect to z gives the differential

equation system that we need to solve

$$\begin{cases}\dfrac{\mathrm{d}^2 f_1(z)}{\mathrm{d}z^2}+\beta_1\left(-f_1(z)^{\frac{3}{2}}+f_2(z)^{\frac{3}{2}}\right)=0\\ \dfrac{\mathrm{d}^2 f_2(z)}{\mathrm{d}z^2}-\beta_2\left(-f_1(z)^{\frac{3}{2}}+f_2(z)^{\frac{3}{2}}\right)=0\end{cases} \tag{S18}$$

where we have defined $\beta_1 = 2\alpha_1/d$ and $\beta_2 = 2\alpha_2/d$. However, Eqs. (S18) is not easy to solve, so we introduce two assumptions for simplification. First, it is assumed that the effective mass of holes is equal to that of electrons. Second, we assume that electrons only reside in one half of the BP film close to the positively charged plate, and holes reside only in the other half, which means

$$\begin{cases}n_1(z)=0, & L/2\le z\le L\\ n_2(z)=0, & 0\le z\le L/2\end{cases}. \tag{S19}$$

Then Eqs. (S18) can be simplified to

$$\begin{cases}\dfrac{\mathrm{d}^2 f_1(z)}{\mathrm{d}z^2}-\beta_1 f_1(z)^{\frac{3}{2}}=0\\ f_2(z)=f_1(L-z)\end{cases} \tag{S20}$$

with the boundary conditions

$$\begin{cases}\left.\dfrac{\mathrm{d}f_1(z)}{\mathrm{d}z}\right|_{z=0}=-\beta_1 d n_0\\ \left.f_1(z)\right|_{z=\frac{L}{2}}=0\end{cases}. \tag{S21}$$

After numerically solving Eqs. (S20), substituting the solutions into Poisson's equation leads to the correction to the potential difference across the BP film

$$\Delta V=\frac{5\varepsilon_{\mathrm{BP}}}{6e}\{A_1[f_1(0)-f_1(L)]-A_2[f_2(0)-f_2(L)]\} \tag{S22}$$

While by still assuming a uniform electric field in the BP film, one can recalculate the QW model detailed in Supplementary Note 2 by treating the screening as an average correction across the QW.

To verify the validity of the TF model, we estimate the density of free carriers that thermal excitation can provide in samples of various layer numbers with screening

considered. The results displayed in Supplementary Table 3 indicate that the thermally excited free carrier densities in relatively thin samples are not sufficient to achieve the density required by the model, and the screening electric field induced by these free carriers is so much smaller than the applied field that it can be ignored. (For the 3-layer BP sample, the screening electric field is five orders of magnitude smaller than the external field. For the 8-layer BP sample, the screening electric field is only 18% of the external field.) For samples with more than 9 layers, thermally excited free carriers are sufficient, and Zener tunneling also serves as a source for free carriers at larger electric fields[11]. (For the 9-layer sample, the screening electric field can reach about 47% of the external field.)

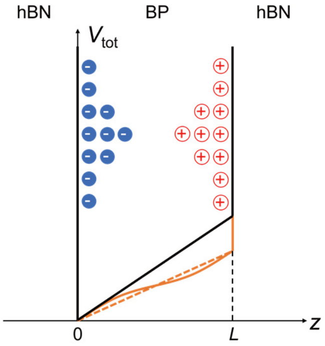

Supplementary Figure 26 | Schematic diagram of the screened QW model. The vertical axis represents the potential energy of electrons. The black thick solid line in the figure represents the one-dimensional infinite QW in an electric field without screening. The orange solid line represents the screened QW model. The orange dashed line represents the approximate potential energy used to revise the QW model.

Layer number	Charge densities Thomas-Fermi model needs (cm^{-2})	Charge densities of thermal excitation at 300 K (cm^{-2})	Ratio of densities could provide versus needed
3	7.85×10^{11}	6.94×10^{6}	8.83×10^{-6}
4	1.13×10^{12}	4.82×10^{8}	4.29×10^{-4}
5	1.48×10^{12}	1.16×10^{10}	7.85×10^{-3}
6	1.79×10^{12}	9.28×10^{10}	5.17×10^{-2}
7	2.09×10^{12}	4.29×10^{11}	0.215
8	2.34×10^{12}	1.13×10^{12}	0.482
9	2.58×10^{12}	2.10×10^{12}	0.812
10	2.79×10^{12}	2.98×10^{12}	1.07
11	2.96×10^{12}	3.55×10^{12}	1.20

Supplementary Table 3 | The calculated thermally excited carrier densities of few-layer BP systems.

Supplementary Note 5

The brightening of the forbidden transitions

According to Fermi's golden rule, with x polarized light traveling in the z direction,

the absorption rate is proportional to the joint density of states and the square of the electric-dipole matrix element[12]:

$$W_{i\to f} = \frac{2\pi}{\hbar}|\langle f|-exE_x|i\rangle|^2 g(\hbar\omega), \tag{S23}$$

where $g(\hbar\omega)$ is the density of states. Since we use the QW model, $g(\hbar\omega)$ is a two-dimensional density in x, y plane and can be treated as a constant. As a result, we only need to consider matrix elements:

$$M = \langle f|x|i\rangle = \int \Psi_f^* (\mathbf{r}) x \Psi_i(\mathbf{r}) \mathrm{d}^3\mathbf{r} \tag{S24}$$

Because the electrons and holes in a QW layer are free to move in the x, y plane but are confined in the z direction, we can write the wave functions in the form:

$$\Psi(x,y,z) = \psi(x,y)\varphi(z) \tag{S25}$$

Thus, we can get the initial and final QW wave functions:

$$\Psi_i \equiv |i\rangle = \frac{1}{\sqrt{A}} u_v \varphi_{\mathrm{hn}}(z) e^{i\mathbf{k}_{xy}\cdot\mathbf{r}_{xy}} \tag{S26}$$

$$\Psi_f \equiv |f\rangle = \frac{1}{\sqrt{A}} u_c \varphi_{\mathrm{en}'}(z) e^{i\mathbf{k}'_{xy}\cdot\mathbf{r}_{xy}} \tag{S27}$$

where A is the normalization area in the x, y plane. The conservation of momentum in the transition requires $\mathbf{k}_{xy} = \mathbf{k}'_{xy}$[12]. As a result, the matrix element M can be divided into two parts:

$$M = M_{cv} M_{nn'} \tag{S28}$$

where M_{cv} is the valence-conduction band dipole moment:

$$M_{cv} = \langle u_c|x|u_v\rangle = \int u_c^*(\mathbf{r}) x\, u_v(\mathbf{r}) \mathrm{d}^3\mathbf{r}, \tag{S29}$$

and $M_{nn'}$ is the overlap for the electron and hole envelope wave functions:

$$M_{nn'} = \langle \mathrm{e}n'|\mathrm{h}n\rangle = \int_{-\infty}^{+\infty} \varphi_{\mathrm{en}'}^*(z)\varphi_{\mathrm{hn}}(z)\mathrm{d}z \tag{S27}$$

Compared with the size of electron, our sample can be recognized as an infinite plane.

As a result, electrons can be treated as nearly free electrons in the x, y plane and M_{cv} is the same for different transitions.

When there is no applied electric field, the envelope wave function in the QW model has an analytical solution. The corresponding integral can be written as:

$$M_{nn'} = \int_{-\frac{L}{2}}^{+\frac{L}{2}} \frac{2}{L} \cos\left(\frac{n\pi z}{L} - \alpha_n\right) \cos\left(\frac{n'\pi z}{L} - \alpha_{n'}\right) \mathrm{dz} \quad \text{where } \alpha_n = \begin{cases} 0, n \text{ is odd} \\ \pi/2, n \text{ is even} \end{cases}. \quad (S28)$$

$M_{nn'}$ is not zero if and only if $n = n'$, as shown in Supplementary Fig. 27a, so the forbidden transitions cannot take place when the applied electric field is zero. When we turn on the electric field, the wave functions in the QW model can be obtained in note 2 and brought to (S27). $M_{nn'}$ is not zero for $n \neq n'$, which means the presence of the forbidden transitions, as shown in Supplementary Fig. 27b. Finally, we can estimate the intensity ratio $I(E_{21})/I(E_{11})$ according to

$$\frac{I(E_{21})}{I(E_{11})} = \frac{|M_{21}|^2}{|M_{11}|^2}. \quad (S29)$$

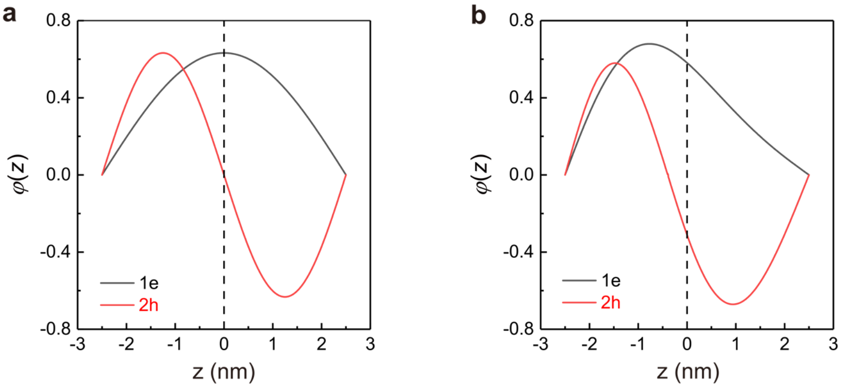

Supplementary Figure 27 | The envelope wave functions for different states in the 10-layer sample without **a)** and with **b)** electric field.

Figure 4e shows calculated intensity ratios at different fields. It has very analogous

trend as the experimental result as shown in Fig. 4d. However, the absolute value is nearly one time smaller. Possible reasons are the following. First, experimentally, the extraction of the intensity ratio has uncertainties, since we have undesirable signals from ice around E_{11} peak area. Second, in the calculation here, we use the effective mass of the bulk rather than the thin film, due to the lack of relevant research on the effective mass of thin films. Finally, the assumption that the electrons can be treated as nearly free electrons in the x, y plane could also contribute to the discrepancy between.

References

1 Deng, B. et al. Efficient electrical control of thin-film black phosphorus bandgap. *Nat Commun* **8**, 14474 (2017).

2 Kumar, P. et al. Thickness and electric-field-dependent polarizability and dielectric constant in phosphorene. *Physical Review B* **93** (2016).

3 Narita, S.-i. et al. Far-Infrared Cyclotron Resonance Absorptions in Black Phosphorus Single Crystals. *Journal of the Physical Society of Japan* **52**, 3544-3553 (1983).

4 Bastard, G., Mendez, E. E., Chang, L. L. & Esaki, L. Variational calculations on a quantum well in an electric field. *Physical Review B* **28**, 3241-3245 (1983).

5 Matsuura, M. & Kamizato, T. Subbands and excitons in a quantum well in an electric field. *Phys Rev B Condens Matter* **33**, 8385-8389 (1986).

6 Pietronero, L., Strässler, S., Zeller, H. R. & Rice, M. J. Charge Distribution incDirection in Lamellar Graphite Acceptor Intercalation Compounds. *Physical Review Letters* **41**, 763-767 (1978).

7 Datta, S. S., Strachan, D. R., Mele, E. J. & Johnson, A. T. C. Surface Potentials and Layer Charge Distributions in Few-Layer Graphene Films. *Nano Letters* **9**, 7-11 (2009).

8 Kuroda, M. A., Tersoff, J. & Martyna, G. J. Nonlinear Screening in Multilayer Graphene Systems. *Physical Review Letters* **106**, 116804 (2011).

9 Castellanos-Gomez, A. et al. Electric-field screening in atomically thin layers of MoS_2: the role of interlayer coupling. *Adv Mater* **25**, 899-903 (2013).

10 Low, T. et al. Plasmons and screening in monolayer and multilayer black phosphorus. *Phys Rev Lett* **113**, 106802 (2014).

11 Zener, C. & Fowler, R. H. A theory of the electrical breakdown of solid dielectrics. *Proceedings of the Royal Society of London. Series A, Containing Papers of a Mathematical and Physical Character* **145**, 523-529 (1934).

12 Fox, M. *Optical Properties of Solids Second Edition* (Oxford University Press Inc., New York, 2010).
